# Accessing and utilising gender-affirming healthcare in England and Wales: trans and non-binary people’s accounts of navigating gender identity clinics

**DOI:** 10.1186/s12913-021-06661-4

**Published:** 2021-06-28

**Authors:** Talen Wright, Emily Jay Nicholls, Alison J Rodger, Fiona M Burns, Peter Weatherburn, Roger Pebody, Leanne McCabe, Aedan Wolton, Mitzy Gafos, T. Charles Witzel

**Affiliations:** 1 Division of Psychiatry, Faculty of Brain Sciences, University College London, London, UK; 2 Department of Public Health, Environments and Society, London School of Hygiene and Tropical Medicine, London , UK; 3 Institute for Global Health, University College London, London, UK; 4 NAM, London, UK; 5 MRC Clinical Trials Unit, University College London, London, UK; 6 56T, 56 Dean Street, London, UK; 7 Department of Global Health and Development, London School of Hygiene and Tropical Medicine, London, UK

**Keywords:** Trans, healthcare experiences, Qualitative research, Mental health

## Abstract

**Background:**

Transgender, or trans, people experience a number of barriers to accessing gender-affirming healthcare and have a range of barriers and facilitators to primary care and specialist services, commonly citing discrimination and cisgenderism playing a central role in shaping accessibility. The pathway through primary care to specialist services is a particularly precarious time for trans people, and misinformation and poorly applied protocols can have a detrimental impact on wellbeing.

**Method:**

We recruited trans participants from an HIV Self-Testing Public Health Intervention (SELPHI) trial to interviews which explored contemporary gender-affirming service experiences, with an aim to examine the path from primary care services through to specialist gender services, in the UK.

**Results:**

A narrative synthesis of vignettes and thematic analysis of in-depth qualitative interviews were conducted with twenty trans individuals. We summarise positive and negative accounts of care under three broad categories: Experiences with primary care physicians, referrals to gender identity clinics (GICs), and experiences at GICs.

**Conclusions:**

We discuss implications of this research in terms of how to improve best practice for trans people attempting to access gender-affirming healthcare in the UK. Here we highlight the importance of GP’s access to knowledge around pathways and protocols and clinical practice which treats trans patients holistically.

## Background

Transgender (or trans) and non-binary individuals are a highly stigmatised and marginalised group, who face barriers to equitable access across many aspects of their lives, including health, employment, education, and family life [1–6]. In particular, trans people face significant barriers in accessing gender-affirming healthcare, often experiencing delays in appropriate referral to secondary and tertiary services, which may be due to primary care physicians’ attitudes towards trans people and their healthcare [2]. Experiences of discrimination and cisgenderism play important roles in the mental wellbeing of trans people. For example, for trans youth, the undermining of gender experiences, experiences of transphobia, and poor interpersonal relationships have been found to increase the risk of non-suicidal self-injury [7]. Cisgenderism refers to various ideologies about trans and non-binary peoples’ genders and/or bodies at an institutional, cultural and social level [8]. This term is being used interchangeably with transphobia, however it’s helpful to distinguish that *transphobia* is the individual’s negative belief and/or attitude towards trans and non-binary people, whereas *cisgenderism* can reflect the cultural, social, and institutional process of transphobia [9].

In the UK, there are various paths trans people might take in accessing gender-affirming healthcare, either through the National Health Service (NHS) or through private General Practitioners (both online and face to face). In England and Wales, medical care is provided free of charge by the NHS. In terms of accessing primary care, one will typically sign up to one GP practice, often within a defined geographical area (with some GP practices having catchment areas), and will remain under their care for the foreseeable future (e.g. until there is a reason to leave, such as relocating to a new area). Under this system, individuals are discouraged from seeing multiple providers and GPs are generally the first point of call for most health issues or concerns. Where referrals are needed for secondary services, usually this will be done by the GP one is registered with. Due to a lack of representative data on trans populations in the UK, it is unclear what proportion of trans people opt to take an NHS route and/or a private route [10]. Although there is some clinic data for trans people who have successfully accessed a Gender Identity Clinic (GIC), referral sources are not highlighted [11].

Here, we discuss the NHS pathway. Guidance on the treatment of trans people describes this as follows: a primary care physician makes a referral to a GIC, where a clinician (usually a psychiatrist) assesses the patient for gender dysphoria (taken from Diagnostic and Statistical Manual of Mental Disorders 5th edition) or gender incongruence (taken from the International Classification of Diseases 11th edition) [12, 13]. Once a gender dysphoria/incongruence diagnosis is reached the patient is referred to an endocrinologist (for hormone replacement therapy), and in some cases to a surgical team for gender affirmation surgeries. While a referral to secondary mental health services is not a pre-requisite for referral to a GIC, the referrals may be made in tandem if there are mental health concerns [14, 15]. The standard of care as outlined by Bouman and Richards (2013) places emphasis on clinicians as gatekeepers in deciding when best to initiate hormones and gender affirmation surgeries and indicates that this can be at odds with trans peoples' desires and needs. More recently information on the guidance given on the care pathway for gender-affirming care by the NHS can be found in their service specification published June 2019 [16]. The specification highlights the pathway noted above, however indicates the ability for patients to self-refer to GICs, in which case they state that the self-referral should not be disadvantaged. The specification also places emphasis on the GICs standards of care, which centres on the role of respect and personal autonomy of people who access gender-affirming services, *in both their gender identity and gender presentation*. The recent specification takes a different approach from the standards of care outlined by Bouman and Richards (2013), instead placing respect, dignity, and equality of trans people as its central guiding principle.

The experiences of trans people accessing GICs vary across different health systems. In Australia, studies examining trans youth and their parents’ experiences of care at paediatric gender services highlight some examples of satisfaction with the service, with parents saying that they would recommend it to others. Examples of practices valued by these parents and young people include understanding the concerns of young people and their families, respecting their thoughts and feelings, and providing encouragement to ask questions and giving information on support networks. These factors were felt to normalise, validate, and affirm the young person’s experiences with gender, and reduce the participant’s distress [17, 18]. In one study from the US, intersecting demographics such as being young, having low income and low educational attainment, lack of private insurance coverage, and experiences of healthcare discrimination have been found to play a significant and often deleterious role in access to and experiences of gender-affirming healthcare services [19, 20].

In the UK context – where this study is based – access barriers to GICs are likely to be similarly multifactorial. Little research to date has investigated the barriers experienced by trans people in the UK when accessing gender-affirming healthcare services. The existing literature has highlighted some specific barriers to GICs, which include inadequate professional knowledge, lack of social support, and fear of discrimination [21, 22]. In addition, there are only eight geographically dispersed gender identity clinics, resulting in most trans people having to travel potentially long distances in order to access care (which, again, may be more or less difficult as a result of, for example, socio-economic circumstances). High rates of dissatisfaction with GIC access has been reported in the UK, particularly in relation to long waiting times. One study of 202 trans participants, recruited in 2012 in the UK, showed that 32 % waited up to three years to access GIC services and 10 % waited more than three years [22]. For non-binary trans individuals, experiences of ‘invisibility’ – such as being overlooked or ignored by services – and managing non-binary identity in a society that largely recognises only two binary genders (male/men, female/women) has resulted in difficulties accessing services (both generic and gender affirming), and their acceptability in meeting the needs of non-binary people, and been shown to increase poor mental health outcomes for this population [23].

While accessing and utilising healthcare services presents significant challenges for many trans people, healthcare providers can also experience barriers in providing good quality and timely care, including lack of knowledge around appropriate care and referral pathways [24]. Where healthcare providers hold prejudicial beliefs about trans people, this will also impact on the provision of good quality care.

This study aims to describe trans people’s experiences on the pathway to and experiences of gender-affirming healthcare and identify key issues and best practice approaches. We do this by exploring accounts of moving from primary care settings to specialist GICs and discussing positive and negative elements of care along this pathway.

## Methods

The SELPHI (An HIV Self-Testing Public Health Intervention) randomised controlled trial (RCT) recruited 10,135 men who have sex with men (MSM) (cis and trans) and trans women who have anal sex with men. Of these 118 participants identified as trans. In order to develop additional nuance of the experiences of trans participants in the trial we conducted a qualitative sub-study with 20 participants. Although this sub-study primarily focused on experiences of and attitudes towards HIVST and sexual health services, we also asked about accessing other primary care and transition related care. This analysis is drawn from the general practice and gender identity service section of the interviews. The SELPHI protocol is published elsewhere [25], as are the results of the RCT trans sub-analysis [26].

Participants were eligible to participate in this qualitative sub-study if they were a participant in SELPHI and identified as trans. Participants were approached only if they had indicated as part of the trial process, they would be happy to be interviewed. Consent was taken in two stages: first the participant was sent an information sheet explaining the study and agreed over email. Second, participants provided verbal recorded consent at the time of the interview.

Topic guides previously used in a study of cis-MSM were adapted by the first and last author. These covered a range of healthcare issues including relationship to general practice, mental health services and transition related care; STI and HIV testing history; HIV/STI testing patterns; and experiences in SELPHI. The study used semi-structured interviews held either face to face (N = 2) or remotely (N = 18). Interviews were audio recorded and transcribed verbatim. The duration of each interview was between 45 min and 1½ hours. Each participant gave informed consent (recorded verbally) and was compensated £30 for their time.

Data organisation and initial analyses were conducted using vignettes. These were created following interview transcribing and were produced by four of the authors (TW, TCW, PW & FB). These narratively summarised participant accounts of their journeys through gender-affirming healthcare. Thematic analysis was conducted by comparing and contrasting findings related to three predominant primary emergent themes relating to aspects of trans health (led by TW and TCW): experiences with primary care physicians; referrals to GICs; and experiences at the GIC. Disagreement on categorisation of quotes and subthemes were resolved using a third reviewer (EJN).

## Results

Twenty trans people were recruited from SELPHI, which included seven trans women with a mean age of 40 years (range 25–57), and 12 trans men with a mean age of 34 years (range 21–54), and one non-binary trans masculine person. In the sample 80 % were white, 45 % had a degree or higher. Three quarters (75 %) of the participants lived in the south of England (south-east, south-west, east, and London). Table 1 presents sample demographics. All but one participant had accessed a GIC and the participant who had not was currently on a waiting list at the time of interview. There was a broad range of experience in terms of when participants had first accessed GICs, with some participants having first accessed GICs very recently, and several having first accessed them 10–15 years ago. In what follows, we present results along the care pathway, initially describing trans peoples’ experiences with GPs, experiences of referral to GICs and, finally, experiences of GICs. Our analysis, presented below, focuses firstly on experiences in primary care, then described participant accounts of referral processes to GICs and finally described experiences of transition related care.

**Table 1:** Study sample

Demographic characteristics		Participant numbers
*N* = 20		
Age	16–25 years 26–40 years 41+	7 (35 %) 6 (30 %) 7 (35 %)
Gender	Trans women Trans men Non-binary	7 (35 %) 12 (60 %) 1 (5 %)
Ethnicity	White Black Latin American Asian Other / mixed Undisclosed	16 (80 %) 2 (10 %) 1 (5 %) - - 1 (5 %)
Sexual orientation	Heterosexual Gay Bisexual Don’t use a term Undisclosed	2 (10 %) 7 (35 %) 6 (30 %) 1 (5 %) 4 (20 %)
Highest educational qualification	Low^1^ Medium^2^ High^3^ Undisclosed	7 (35 %) 3 (15 %) 9 (45 %) 1 (5 %)
Randomisation	RT BT nBT Not randomised	4 (25 %) 10 (50 %) 5 (25 %) 1 (5 %)

### Experiences with primary care physicians (GPs)

Participants described various experiences with their GPs, with some having positive experiences and supportive relationships with their healthcare provider, and others feeling that their care was lacking or unsupportive. Accounts of positive experiences with GPs typically revolved around the provision of care which was felt to be appropriate to their experience, was responsive and which treated the patient as a whole person. Being treated as a ‘whole person’ meant not being reduced to one element of their experience, such as their trans identity. Negative experiences were often perceived to arise from GPs’ lack of knowledge or experience of engaging with trans patients. Some participants experienced care in which their trans identity was seen as either a distraction, cause, or obstacle to receiving basic care. Barriers to good care were also evident when GPs had a negative reaction to disclosure of gender.

Some participants reported having paid to access private GPs specialising in trans health (sometimes online), allowing quicker access to transition related healthcare. This included accessing bridging prescriptions of hormones prior to being placed on a waiting list for specialist services:

“… once I had seen them [GIC] and got assessed I had to wait a year to actually get anything from them so that was less good, but in the meantime, I was waiting I went to a private GP and got hormones.” PN20 (aged 49, trans woman).

Although it may be assumed that such divergences from standard practice might complicate or cause problems in accessing care elsewhere, some interviewees described their GPs attempting to work around and respond to their needs, including the prescriptions they had acquired privately. For example, the interviewee quoted above went on to describe an NHS GP taking on the responsibility of prescribing hormone replacement therapy which had initially been accessed online. Speaking about their GP, they recounted:

“They have been … they’ve been absolutely amazing. They really have. When I managed to get onto the hormones with the online clinic, and I was doing my blood tests with my own GP […] and I said, “Look, you know, if you possibly could prescribe?” And she said, “I don’t know. I’ll look into it.” And that took several months, going backwards and forward, but eventually she came back and said, “Yeah, I apparently can, the only thing we can’t give you is the creams to slow down hair growth.” And I thought, “That’s not really a problem, I’m getting laser, so…” So, they then took over prescribing and they’ve been brilliant.” PN20 (aged 49, trans woman).

Although this back and forth took longer than may have been desired, the interviewee’s GP not only took on this responsibility, but also demonstrated a *willingness to see what was possible*, while also pre-empting other kinds of treatment that may be sought by their patient. While for the interviewee above, *good care* involved being listened to, and the GP having a willingness to learn and find out what might be possible in terms of treatments. For others, good care came in the form of experience of and knowledge about treating other trans people. As PN17 below describes:

“So, I picked the practice and luckily, I’m not the only trans person there, they know what they’re talking about. They’re very confident with hormones, they’re not constantly going, “You’ve got to go back to [GIC], you’ve got to do this, you’ve got to do that.” […] I’ve done it before; they know how to adjust my levels and things like that. […] But I’ve been very, very lucky with my GP, very lucky, they have been so good. I’ve seen like four or five different GPs at the practice and I know which ones to sort of go to for certain things because they’re just better at understanding that” PN17 (aged 22, trans man).

This experience also speaks to a need to actively navigate healthcare services, as even though PN17 has found a GP practice that meets their needs, they still need to request the GP most experienced in managing the needs of trans patients.

Across the interviews there was also a broader appreciation of the value of GPs expertise and an ability to respond to the needs of their patients. This skill and knowledge may come from their training. However, having pre-existing knowledge was not the only way in which GPs were able to deliver good care. That is, this was not only in relation to what a GP *knew already*. It was also important that a GP appreciated the importance of language and was willing to follow the lead of their patient. As PN14 explained:

“He’s followed my own language, about how I would describe my body, he’s used the same language, and that kind of stuff is so important I think.” PN14 (aged 48, trans man).

Here, good care is expressed as a sensitivity to the importance and subtleties of language, as well as a willingness to take the patient as an expert in their own body and experiences. Elsewhere across the interviews, good care was expressed in a variety of ways. As PN09 below describes, this includes an attentiveness to the whole person and the overlapping elements of health and wellbeing in relation to, but also beyond, gender transition:

“It was that she just gave a shit which is the most important thing. And she put me on [named antidepressant] which was new. New for me and not a thing I’d ever expected to take. But it helped enormously. And she kept a really close eye on me. Remembered what I’d said to her in previous appointments. I never felt rushed. Yes, she was really good. Just all the pastoral stuff, really, that really matters. And once I went on T[estosterone] and had different things like a skin condition at one point, she wasn’t blind to how my mental health and my transition and other aspects of my health interacted. And she saw me as a whole person. It was a holistic experience.” PN09 (aged 26, trans man).

In stark contrast, some participants described experiences with GPs where this had not been the case. For example, PN15 discussed an experience in which he felt that his healthcare provider was only able or willing to take into account one element of his experience at a time:

“It’s very frustrating because everywhere you go, they want to bring up the trans thing, literally everywhere. Or if they don’t want to bring up the trans thing, they want to bring up like … because I have [health condition] and they want to bring that up every time and then somehow pull out all these trainee physicians out of nowhere. And you’re just like, “Can I just have a normal appointment without a million people appearing in the room?” PN15 (aged 51, trans man).

While PN15’s experience above was of healthcare providers either focussing only on his trans status, or completely avoiding it, PN11 below describes how her GP was so shocked at her disclosing her trans status that she didn’t feel able to take it further:

“It took years longer than what it should because with my first GP, you know when I was broaching the subject and stuff and they were flabbergasted and I was just like, “Oh, it doesn’t matter,” you know, and just left it” PN11 (aged 30, trans woman).

Breaking through the initial barrier of having to disclose one’s trans status to a GP may take a long time, and first attempts may be undermined by GPs who are not experienced in treating trans patients. While earlier we described positive experiences with GPs as either having prior knowledge or training on trans issues or a willingness to learn, these negative experiences further underscore the importance of GPs’ education on trans health issues, collaborative medicine, of taking trans patients as a *whole person* into account, and of the negative effects of poor care.

### Referrals to GICs

Positive accounts of acquiring a referral to a GIC arose from experiences with well-informed GPs with a good awareness of pathways and processes. However, some participants reported that gaining a referral to specialist gender services was fraught with difficulty. The experience of waiting was profoundly difficult for participants, made more so with inaction on prompt, and timely, referrals being sent to GICs. Participants also reported a lack of clear information about the process itself that can be easily accessed when requesting a referral.

Participants also described GPs who were poorly informed about pathways and referral processes and having undergone unnecessary interim referrals to mental health services. Where participants had positive experiences, this was most often in the form of straightforward and relatively frictionless referral processes which avoided these additional complications, and in which individuals received good care.

Echoing some of the experiences described in the previous section, PN01 described how, although her GP had never made a GIC referral before, he had taken the time and effort to find out what he needed to do and, as a result, she was granted timely access to services:

“He turned round and he sorted everything I needed, including writing a report and sending me to where I needed to go. Although he had never done it before, he made the effort to find out all the pathways and everything, where I have to go and what I have to do. He gave me all the advice he could” PN01 (aged 56, trans woman).

For some, this process was not nearly as smooth. Where little was known about the process beforehand, interviewees accounts highlighted how asking their GP for a referral required what can be understood as a significant leap of faith, including in relation to concerns about whether they would receive a positive response from their GP. As PN06 describes:

“I think I was nervous ‘cause there wasn’t much information available about what the process was like and then you don’t know what they’re going to say to you. If they’re going to agree with you. But even the people, even though they’re… you kind of worry. And then obviously the long wait.” PN06 (aged 28, trans man).

Here, not knowing what the referral process would look like left PN06 feeling unable to anticipate his GP’s response to his request. While this may be a barrier to acquiring a referral, PN02 below describes how, even once this initial barrier is overcome, asking for a referral is not necessarily a one-time event and may require several attempts, adding more time to the wait for specialist services:

“Oh, it was awful. I went three times to my GP before I actually got the referral and even then, I didn’t trust so I ended up having to ring the GIC just to confirm it. ‘Cause I’d asked for the referral two or three times but the first time they just didn’t do it.” PN02 (aged 24, trans man).

As well as raising significant questions with regards to the need PN02 felt to follow up and ensure that a referral had been sent as promised, PN02 attributed this situation to his healthcare provider’s lack of knowledge about the protocol for referring patients to the GIC:

“I honestly just didn’t… I don’t think they actually knew what the protocol was. I think they were just guessing. I think the first time I just don’t think I was clear enough that that’s what I wanted. The second time, obviously, they tried to go a different way about it, saying I needed to see a mental health person first. And then the third time, I just basically came in with all of the information. Told them what to do.” PN02 (aged 24, trans man).

On all three occasions that PN02 had tried to get a referral, different requirements had been placed on him, a situation he attributes to his GP’s lack of knowledge about protocol.

All participants discussed an initial wait for secondary mental health services (most often, a psychiatrist) prior to the further wait for GIC services. Before a referral to a GIC was to be initiated, for example, PN16 experienced delays in referral due to the requirement to see mental health professionals beforehand. This requirement was imposed by his GP unnecessarily due to what PN16 perceived as a lack of knowledge:

“He didn’t realise, actually, at the time that he could refer straight away. So, he referred me to the mental health services. And I saw a psychologist and he said, oh, your GP could have just directly referred you” PN16 (aged 51, trans man).

Not just a matter of inconvenience, long waits for referrals to be processed can have significant detrimental effects. For example, below PN01 describes the profound impact on their mental health and their consequent responses to poor care and lack of access:

“Eventually I tried to castrate myself and the hospital turned around and said, “No, you’re nuts,“ and sent me to a psychiatrist, which then sent me to the gender clinic. If my GP had done stuff, it would have been a lot easier process if you know what I mean. PN01 (aged 56, trans woman).

These experiences demonstrate the need for timely referrals, especially to GICs, where long waiting times are likely to be experienced. The above shows the need for many trans people to ensure their referral has been secured and processed. This may amplify the anxiety surrounding the probability of a long wait time. Whilst willingness to learn and educate oneself is a sign of good practice, better care quality needs to mitigate anxieties experienced by trans people through strengthening awareness and education on referral procedures for all healthcare providers.

### Experiences at the GIC

Narratives of quality care at GICs revolved around a feeling of confidence that participants were understood and believed by healthcare providers, and that they were not subject to unnecessary referrals (predominantly to mental health services, as previously discussed). Good care was thus expressed primarily as a matter of everything ‘running smoothly,’ and of a feeling that GIC healthcare providers were understanding of their needs and of process and protocols. Negative experiences often described the expectations of GIC healthcare providers that patients should conform to dominant narratives regarding the gender binary (in hyper-feminine or masculine manner) in order to access gender affirming support. This led to participants both feeling that they were not being fully understood, and also feeling that they had to perform gender in particular ways in order to receive good care. This need to perform gender in ways felt to be more accepted by GIC healthcare providers also had the effect of precluding the possibility of these providers fully understanding their patients, which may have also led to a more nuanced understanding of gender expression and identity.

Positive experiences tended to include participants feeling they were able to be open about their sexuality, gender expression, and gender identity without being doubted or needing to present themselves in particular ways to get the care they needed. An example from PN18 exemplifies what a positive experience might look like, and in which he felt heard, cared for, and confident in the care that he was receiving:

“I felt very heard and very believed and like I was in very capable hands. Like it was still a long process in terms of I had to wait a couple of years for waiting lists to go down. But it wasn’t so long in terms of complications, it would just be like a couple of appointments they’ve heard what they needed to hear and now they can pass me on to the relevant services. So yeah, overall just had a really positive experience with [GIC], and doctors were very understanding, again, none of them tried to like equate my transness with my mental illness.” PN18 (aged 20, trans man).

Negative experiences of GICs were often, at least in part, the result of normative assumptions of healthcare providers regarding gender and sexuality. For some, this was a result of requirements of having already made a ‘social transition,’ prior to medical and/or legal transition [27]. Also referred to as ‘real life experience’ or the ‘real life test,’ this requires that a trans person lives in their affirmed gender role for a period of time before being able to access services [28]. Below, speaking of their experience over 15 years earlier, PN05 highlights how this process was often also informed by narrow views of gender-appropriate styles of dress and presentation:

“Yeah. Because at that time, the gender identity clinics were still following quite a cis-normative process where you have to dress looking like a man, you know, you couldn’t be fluffy. The women had to dress like women and look like women and look like they’d be successful passing as women, and it was very much like that then.” PN05 (aged 52, trans man).

As well as having to dress in ways felt to conform to healthcare providers’ assumptions regarding gender expression, trans people may share with them the kinds of stories felt most likely to result in the care they need. This also has the negative effect of obscuring the full picture and spectrum of trans lives for the medical practitioners responsible for their care:

“At the time, it was very much known that you didn’t talk about being gay, so, all trans-people at that time shared what their experiences were, so basically all the gender-identity clinic that were getting was the same trans story from every trans person because we knew that worked, they weren’t actually getting a picture of what real trans lives were like. But I know that’s changed gradually over the years as they’ve realised, and that we were just telling them… Basically I just told them what they needed to hear, because I knew what I needed.” PN05 (aged 52, trans man).

PN09 similarly described how they had learnt to behave and present themselves in ways which would be more likely to align with the assumptions of their healthcare providers and, as such, would increase their chances of receiving the care they needed. Referring to this as a matter of gatekeeping, PN09 describes:

“They’re not care providers. They’re gatekeepers. So, they’re just… You go up, you get asked, are you sure? Are you sure? Are you sure? And then they green light hormones and surgery and whatever. When I needed to get stuff from them, I would be very masculine for them and there’s certain things I don’t tell them and certain things I emphasise”. PN09 (aged 26, trans man).

These normative assumptions about gender further hindered access for non-binary people. As such, some participants who were non-binary chose not to disclose this to their healthcare providers due to fears of compromising care. This was sometimes experienced as a difficult balance. As PN08 (23-year-old non-binary trans man) explained this, they felt comfortable being open about their fondness for wearing make-up and feminine clothes but stopped short of disclosing their non-binary identity. As they put it, “I just didn’t want to push it too far.” Moreover, unsure that it was necessary to hold back this aspect of their identity, they did so anyway:

“I don’t think I discussed being non-binary with them ever, I don’t think.… I’m… I don’t know if that was… that I actually had to do that, but I just did that because I didn’t want to compromise my care.” PN08 (aged 23, non-binary trans man).

Unsure of whether it was necessary, the risk of having their care compromised was too great. It should be noted that this was not a matter only of disclosure, but also a felt sense of being observed:

“One thing I’ve noticed as well in appointment letters, and it’s happened with the psychiatrist as well, they use your mannerisms and your body language as evidence of your gender” PN07 (aged 23, trans man).

These experiences clearly demonstrate the impact of normative assumptions regarding gender, and also sexuality, within the GIC. This might be experienced as a pressure to withhold information, to perform gender in particular ways, or as a strong sense of being the object of surveillance.

## Discussion

In this paper, we have described both the positive and negative experiences of trans people attempting to access gender-affirming healthcare in the UK. The majority of trans participants reported GPs as their first point of call in their attempts to access gender-affirming healthcare. Here, experiences were mixed, with some participants describing attentive and knowledgeable GPs who provided affirmative and compassionate care, and others feeling their GPs were inexperienced and unfamiliar with trans issues. Recent findings on the Trans Pathways project in Australia highlight this as a common issue, with many clinicians lacking knowledge and expertise on trans issues (particularly trans youth) [29]. GPs having prior knowledge and familiarity with trans issues, including care pathways, was also significant in attempts to secure referrals to GICs. Here, many participants described their experiences of being subjected to unnecessary referrals to mental health services. These experiences may reflect a lack of awareness of appropriate referral pathways, whereby mental health services were required to assess a patient prior to being referred to GICs. In some circumstances a referral to mental health services is warranted, particularly where depression, anxiety, and/or suicidality is presented, however best practice would advise making this referral simultaneously with a referral to a GIC so as not to introduce additional and unnecessary waiting times [14]. There are still gaps in the education of GPs on how best to treat trans patients [30], and work conducted by Bauer et al. (2019) highlights the impact of institutional and informational erasure on the healthcare access of trans individuals [31]. While our research has highlighted the potentially deleterious effects of poor care, convoluted care pathways and long waiting times, it has also made evident a willingness on the part of some GPs to offer the best care they are able, even if this means having to search out information which is not known already or readily available.

This analysis has also underscored the significant amount of knowledge and skill required for trans people to effectively navigate care pathways and the sometimes profound effects of long waiting times on wellbeing.

Indeed, for some, disclosing one’s trans identity and asking for the initial referral was a significant barrier to care. Some participants, on the other hand, seemed to have greater ease in navigating what may be experienced as labyrinthine processes of accessing care: by taking things into their own hands and accessing prescriptions online, knowing which GP was most likely to be understanding, or being adept at presenting oneself in ways thought more likely to result in good care. This is reflected in the current literature on patient experiences in primary healthcare. Willis et al. (2020) reported on older trans peoples experiences (aged 50–74) and highlight trans patients as reluctant educators, whereby gaps in the knowledge of GPs are filled by patients [32]. Other studies have highlighted this concern amongst other generations of trans people, particularly amongst trans youth, and from physician perspectives [24, 33]. Navigating healthcare services, securing referrals and accessing good care should not require specialist knowledge or skill. Waiting times were also a significant issue for most participants, reflecting other studies on trans health [22, 34–36]. Current estimates on accessing adult services is two to three years from the point of a formal referral being accepted to a first appointment with a psychiatrist [34]. Our analysis has echoed this, as well as underscoring the significant impacts waiting times can have on the wellbeing of trans people. We found that participants may withhold information or use body language/mannerisms to assuage provider concerns and reduce the risk of comprising access to care. This is reflected in the literature, whereby a large proportion will withhold information such as employment status, concurrent mental health concerns, and sexuality, as these are often seen as a hindrance to accessing care [22].

Overall, a recurring theme in our analysis has been the importance for trans people of being treated holistically rather than being reduced to gender identity and expression alone. While this concern is carried throughout, nowhere is this clearer than in the experiences of trans people accessing care at GICs. Although we may have expected experiences to have been more positive in specialist services than in GPs, many participants spoke of normative assumptions about gender presentation hindering their care, these accounts are both historical and recent. Previous work supports our findings here, highlighting the gate-keeping role of those in charge of gender-affirming healthcare [22, 29], and goes against the standards of care highlighted in the NHS’ service specification, which promotes respect of service users’ gender identity and gender presentation [16]. This underscores the importance of ensuring that patients are better able to convey their needs and experiences to their healthcare provider, as more expansive understandings of gender presentation being employed within these services, will likely result in better care for trans patients. Carlile (2020) highlights what this expansion could entail, first there is a need to educate and train trans healthcare providers as the fear of discussing one’s gender identity and expression as being fluid is linked to the struggle of accessing gender affirmative healthcare, particularly for non-binary youth. Secondly, decentralising care for trans youth particularly, and arguably trans people of all ages, allows more accessible and quicker access to gender affirming medication, and where an emphasis away from rigid thinking around binaries and towards an expansive and nuanced approach to gender identity and expression can allow non-binary people to receive the care they need.

Although there were positive experiences of GICs within the accounts offered here, many of our interviewees described the GIC as a space in which gender expression was policed as well as a feeling of being observed. This is concerning, especially in the sense that it may have the effect of obscuring the complexity of trans lives and experience from healthcare providers, as patients learn to present themselves in ways felt to be conducive to good care. These issues will likely be especially pronounced for people whose gender does not fit into the male/female binary. Experiences in GICs as well as positive experiences with GPs have highlighted the importance of gender-affirming care and of treating trans people as experts in their own bodies and experiences [37, 38]. Our results indicate this is often not the case, especially in GICs.

### Limitations

This qualitative enquiry of the experiences of trans men and trans women provides unique insights into quality of care in the UK context. Our diverse sample highlights a range of experiences from across the age spectrum and from a group with a range of educational qualifications. Nevertheless, some limitations are noted. The SELPHI trial in which this sub-study was embedded included only trans men and trans women who have sex with men. During the interview process, one individual discussed being non-binary, however non-binary people were not specifically included in recruitment, nor were trans people who only have sex with women, or indeed those who selected woman who have sex with men, thus potentially screening out an eligible population. Experiences of seeking and engaging in care may vary across this spectrum, especially for non-binary people who face specific issues in accessing care including, but not limited to, erasure of their gender identity [23]. Future research needs to engage with non-binary people on their experiences with the process of accessing gender-affirming healthcare. It should also be noted that individuals who took part in SELPHI had an interest in accessing HIV self-testing. Groups most likely to self-test tend to do so because they face pronounced barriers to testing services including shame, fear and stigma [39]. It may be that some participants experience these same barriers when considering accessing other services, perhaps including GICs. This study was majority White and therefore does not reflect the experiences of Black, Asian and minority ethnic trans people. Future research needs to examine the experiences of trans and non-binary people of colour and the intersections of race, gender, and sexuality and the experiences of accessing gender-affirming healthcare.

### Strengths

A key strength of this study is the focus on the path from initial contact with primary care services through to experiences at the GIC. This enables access to a clearer picture of the whole journey through services, rather than focussing on only one element of this. This has allowed us to identify where improvements can be made in the provision of gender-affirming healthcare, as well as common threads throughout the whole journey.

This study has included an engagement with both recent experiences as well as those occurring 10–15 years ago. As previous experiences are likely to have an influence on engagements with and experiences of healthcare provision now, this is a key strength of the study. It has also allowed us to examine potential changes in service experience over the last few years, although unfortunately we have found little evidence of trans people experiencing better quality of care in more recent engagements, therefore underscoring the need to translate evidence into practice.

## Conclusions

This study has explored the experiences of trans and non-binary people in accessing gender-affirming healthcare throughout the care pathway: from first disclosure of gender to a primary care physician, to obtaining a referral to a GIC, and finally to experiences at a GIC. These accounts have included descriptions of both good practice and also practice in need of improvement. It has highlighted the importance of both providing affirmative support for trans men and trans women and reducing gatekeeping by recognising patient expertise. We have highlighted a need for further practical guidance to ensure affirmative practice is followed, and that more research is needed on how best to support trans men and trans women as they engage with long waiting times.

## Data Availability

The data that support the findings of this study are available on request from the corresponding author, TW. The data are not publicly available due to the risk of identifying the participants and therefore compromising their right to anonymity and safety.
